# Specific Blood Plasma Circulating miRs Are Associated with the Physiological Impact of Total Fish Meal Replacement with Soybean Meal in Diets for Rainbow Trout (*Oncorhynchus mykiss*)

**DOI:** 10.3390/biology12070937

**Published:** 2023-06-30

**Authors:** Francisco Javier Toledo-Solís, Ana M. Larrán, Juan B. Ortiz-Delgado, Carmen Sarasquete, Jorge Dias, Sofia Morais, Ignacio Fernández

**Affiliations:** 1 Aquaculture Research Center, Agro-Technological Institute of Castilla y León (ITACyL), Ctra. Arévalo, Zamarramala, 40196 Segovia, Spain; fj.toledos@gmail.com (F.J.T.-S.); ita-largaran@itacyl.es (A.M.L.); 2 Consejo Nacional de Ciencia y Tecnología (CONACYT), Av. Insurgentes Sur 1582, Col. Crédito 6 Constructor, Alcaldía Benito Juárez, Mexico City 03940, Mexico; 3 Instituto de Ciencias Marinas de Andalucía-ICMAN/CSIC, Campus Universitario Río San Pedro, Apdo. Oficial, Puerto Real, 11510 Cádiz, Spain; jbosco.ortiz@csic.es (J.B.O.-D.);; 4 SPAROS Ltd., Área Empresarial de Marim, Lote C, 8700-221 Olhão, Portugal; jorgedias@sparos.pt; 5 Lucta S.A., Innovation Division, UAB Research Park, 08193 Bellaterra, Spain; sofia.morais@lucta.com; 6 Centro Oceanográfico de Vigo, Instituto Español de Oceanografía (IEO), CSIC, 36390 Vigo, Spain

**Keywords:** liquid biopsy, digestive system, non-coding RNAs, miRNAs, transcriptomics

## Abstract

**Simple Summary:**

To warrant food security and nutrition, more sustainable aquafeeds should be used in fish farming, and the content of fish meal (FM) in fish diets should be reduced. Soybean meal (SBM) is one of the most commonly used alternative raw materials to replace FM in commercial diets, although a high SBM dietary content can affect fish growth, physiology, and wellbeing. Although these effects are known to be produced by several antinutritional factors (ANFs), the underlying mechanisms are still not fully understood. Deeper characterization of how SBM dietary content alters fish physiology will allow the development of more integrative fish physiology monitoring. In the present work, the expression of six microRNAs (miRs) in blood plasma was associated with a high SBM dietary content and related physiological consequences. Circulating miRs can be used as a less invasive procedure to monitor fish physiology without the need of animal sacrifice, allowing the development of more sustainable aquafeeds with the use of fewer animals in experimentation.

**Abstract:**

High dietary SBM content is known to induce important physiological alterations, hampering its use as a major FM alternative. Rainbow trout (*Oncorhynchus mykiss*) juveniles were fed two experimental diets during 9 weeks: (i) a FM diet containing 12% FM; and (ii) a vegetable meal (VM) diet totally devoid of FM and based on SBM (26%). Fish fed the VM diet did not show reduced growth performance when compared with fish fed the FM diet. Nevertheless, fish fed the VM diet had an increased viscerosomatic index, lower apparent fat digestibility, higher aminopeptidase enzyme activity and number of villi fusions, and lower α-amylase enzyme activity and brush border integrity. Small RNA-Seq analysis identified six miRs (omy-miR-730a-5p, omy-miR-135c-5p, omy-miR-93a-3p, omy-miR-152-5p, omy-miR-133a-5p, and omy-miR-196a-3p) with higher expression in blood plasma from fish fed the VM diet. Bioinformatic prediction of target mRNAs identified several overrepresented biological processes known to be associated with high dietary SBM content (e.g., lipid metabolism, epithelial integrity disruption, and bile acid status). The present research work increases our understanding of how SBM dietary content has a physiological impact in farmed fish and suggests circulating miRs might be suitable, integrative, and less invasive biomarkers in fish.

## 1. Introduction

Aquaculture long-term sustainability is the main goal to improve nutrition and food security for a growing human population [1], and relies, at least partly, on decreasing forage fish usage [2]. During the last decades, superior feed efficiency and fish nutrition, as well as a lower fish-in–fish-out ratio, have been attained for all fed species. Indeed, a reduction in the dependence on marine ingredients was translated into an increasing reliance on terrestrial ingredients (reviewed in [3]). Despite the improved implementation of alternative oil sources in aquafeeds [4,5], fish meal (FM) replacement with alternative protein raw materials still has a margin to be increased (from 50 to 90%) independently of the different plant sources tested [6]. In this sense, greater knowledge of the physiological impact they might have on fish species and the identification of suitable biomarkers are urgently required.

Meal from insects and other invertebrates, microorganisms (single-cell protein), animal byproducts, and vegetable origin (legumes and macro- and micro-algae) have been tested as alternative raw materials to replace FM, with different factors limiting the degree of substitution in commercially available feeds [7,8]. Among them, FM replacement is still majorly based on protein sources of vegetable origin, with the achieved % depending on the fish species considered. Soybean meal (SBM) is, by and large, the most commonly used protein source in animal feeds due to its high protein content and “favorable” amino acid profile, among other factors such as worldwide availability and a low price [9]. However, even when pre-treated (heat), amino-acid-supplemented, and/or defatted SBM is used, a high SBM content has been reported to have a deep impact on fish physiology. Increased mortality, decreased growth, feed intake, energy and fat digestibility, induction of an inflammatory response in the distal intestine (enteritis), and/or an increased sensitivity to diseases, as well as an altered microbiota, have been shown as the main effects of high FM replacement with SBM [6,10]. These effects have been related to potential deficiencies in particular micronutrients like vitamins, amino acids and/or minerals and/or to the presence of proteinase inhibitors, lectins, phytic acid, saponins, phytoestrogens, antivitamins, phytosterols, and/or allergens in SBM [6,11,12]. 

In order to increase the % of FM replacement with SBM (or any other alternative raw material), a deep and wide knowledge of the specific molecular pathways underpinning their impact on fish physiology is crucial. Different genomic approaches have been applied to analyze particular and key tissues in a diverse set of fish species. In salmonids, previous studies using targeted (quantitative polymerase chain reaction (qPCR)) approaches identified particular genes whose expression was altered by high SBM dietary content, most of them involved in cholesterol and bile acid biosynthesis and transport as well as in lipid metabolism [13]. Later, studies applying wider (microarray or RNA sequencing (RNA-Seq)) approaches revealed that immune response, lipid metabolism, proteolysis, transport, metabolism and detoxification, purine metabolism, cell proliferation, and/or apoptosis, among other processes, were also transcriptionally altered in the liver and distal intestine [14,15]. Despite the wider picture offered by these omic approaches, the precise mechanism by which SBM-induced enteritis occurs is still not fully understood.

Among the DNA sequences actively transcribed, non-coding RNAs (ncRNAs) have a regulatory role over gene expression, with microRNAs (miRs or miRNAs) as the largest and most widely characterized population (reviewed in [16,17]). The most commonly recognized function of miRs is to regulate mRNA translation through the binding of their seed region (2–8 nt from the miR 5′ end) to the 3′ UTR (most commonly), 5′ UTR, or coding region of the target mRNA. The biogenesis and functioning of miRs, as well as the steady increase in literature showing the role of miRs (and other ncRNAs) behind a diverse set of biological functions of economic and ecological interest in fish species, were recently reviewed in [18,19]. For example, the hepatic expression of different miRs was related to the physiological response toward the lipid content in diets for Atlantic salmon (*Salmo salar*; [20]) or the full replacement of FM and FO with plant sources in rainbow trout (*Oncorhynchus mykiss*) diets [21]. More specifically, some hepatic long ncRNAs in rainbow trout were associated with strains differing in plant-protein diet tolerance and seemed to be involved in lipid metabolism and immunity responses to SBM [22]. Moreover, a high (50%) FM replacement with SBM in diets for hybrid grouper (*Epinephelus fuscoguttatus* ♀ × *Epinephelus lanceolatus* ♂) led to a differential expression of 92 miRs in the intestine, and most of them also were involved in fish immunity [23]. 

Deciphering genomic, transcriptomic, proteomic, and/or metabolomic signatures at the tissue level offers extremely important information to understand and unveil the long-term potential risk and physiological consequences for farmed fish when using a particular raw material in aquafeeds. For example, the liver transcriptome includes gene expression responses more related to overall growth/health performance, while the intestinal transcriptome reflects specific and direct responses to SBM [14]. In contrast, blood plasma miRs are of particular interest because they might provide further insights on how dietary interventions affect fish physiology in a more integrative manner, while at the same time they represent a more ethically (reducing the number of animals used in experimentation) and statistically (reducing the interindividual variability) suitable fish monitoring procedure due to the non-lethal sampling technique [18]. In this regard, interesting features of mature circulating miRs include: being actively released from cells to different biofluids, being very stable under different conditions, and representing another system of cell–cell communication [24]. Indeed, the use of high-throughput analysis of fish liquid biopsies already allowed the identification and quantification of circulating miRs as a less invasive procedure to monitor fish physiological status [25,26,27,28]. Moreover, through bioinformatic analyses, mRNAs targeted by miRs can be predicted [18]. Although these computing analyses still require further improvements, the first evidence of the potentially altered pathways might be obtained, and a reduced list of mRNA–miR interactions can be prioritized for experimental validation [18].

In the present study, we aimed at the characterization of the effects of total dietary FM replacement with high SBM content in rainbow trout using current feed formulations applied at the industrial level (maximal FM and FO contents limited to 12 and 7%, respectively). Fish growth performance, feed apparent digestibility, activity of digestive enzymes, and histopathological analysis were first evaluated. We additionally hypothesized that this physiological impact might be reflected in the population of circulating miRs from blood plasma and that the identification of differentially expressed circulating miRs could provide key knowledge on the molecular mechanisms behind high SBM dietary content through the bioinformatic prediction of targeted mRNAs.

## 2. Materials and Methods

### 2.1. Ethical Statement

All experiments complied with the ARRIVE guidelines [29] and were performed according to section 2010/63/EU of the European Parliament and Council and guideline 86/609/EU of the European Union Council and Spanish legislation (RD53/2013) in order to warrant an ethical animal experimentation as well as fish welfare. The people involved in the experiments were qualified to handle animals for experimentation according to Order ECC/566/2015 from Spanish legislation. All procedures were previously approved by the Bioethical Committee of ITACyL in order to fulfill the administrative requirements prior to conducting the planned research (approval number: 2018/35/CEEA).

### 2.2. Experimental Diets

Two experimental diets were specifically formulated to be isonitrogenous, isolipidic, and isoenergetic, containing 12% (FM diet) or 0% of FM (VM diet) and supplemented with lysine and methionine to fulfill the nutritional requirements of rainbow trout (Table 1). Feeds were manufactured by Sparos Lta (Olhao, Portugal) and extruded in appropriate size pellets (2 and 3 mm).

### 2.3. Experimental Design and Rearing Conditions

A total of 2000 all-female rainbow trout eggs were shipped from Mundova (PISCIFACTORÍA RÍO MUNDO SLU., Albacete, Spain) to the experimental facilities of the Aquaculture Research Center (ITACyL, Segovia, Spain). The eggs were incubated and the hatched embryos reared until reaching 20 g (approx. mean wet body weight (WBW)). A total of 240 fish were acclimatized for 15 days before the experimental trial, after which they were randomly allocated into 8 cylindrical fiberglass tanks (500 L) connected to a recirculating aquaculture system. A total of 27 animals with an average and standard deviation value of 23.23 ± 0.05 g wet weight and a 12.48 ± 0.02 cm total length (TL) were allocated in each tank. The experimental diets were tested in quadruplicate. Fish were hand-fed to apparent satiation once (between 8:00 and 9:00 h) a day (until a maximum of 3% daily feed intake) for 9 weeks.

During the growth trial, the water temperature was maintained at 14.5 ± 0.3 °C, the dissolved oxygen was maintained at 8.1 ± 0.3 mg/L, and the specimens were reared under a 12:12-hour light:dark photoperiod cycle. The ammonium and nitrite water concentrations were monitored daily to keep them below toxic values (0.01 and 0.05 ppm, respectively).

### 2.4. Fish Sampling and Growth Performance Assessment

Fish were sampled every 21 days for WBW and TL measurements in order to adjust the daily feed ration. Fish were lightly anesthetized with MS-222 (80 mg/L). TL was monitored using a graduated ichthyometer (±0.1 mm) and WBW with a GRAM S3R-6KD balance (±0.1 g). Every day, mortality and feed intake in each tank were recorded. Feces were collected in a settling column during the last two weeks of the trial for apparent digestibility analysis. For this, after trout feeding, the tanks and settling columns were cleaned. Feces were collected the next morning.

At the end of the trial, 3 fish from each tank were randomly sampled and euthanized with an overdose of MS-222 (300 mg/L) after 24 h post-feeding to collect blood (for isolation of circulating miRNAs) from the caudal vein and dissected for viscero- and hepato-somatic indexes. Specific proximal (after the last pyloric caeca) and distal (after the enlargement of the intestine) sections of the intestine (for histological assessment) as well as fillet samples (for proximal composition) were also taken from the same fish. The whole digestive system (for enzymatic analyses) was taken from another 3 fish from each tank after 12 h post-feeding. Growth performance indexes were calculated as previously described [30]. Daily feed intake (DFI) was calculated as follows:

*DFI (%BW/day) = [Ingested food/((IBW + FBW) × 0.5 × T)] × 100*

where *BW* is the body wet weight, *IBW* is the initial BW, *FBW* is the final BW, and *T* is the time in days.

### 2.5. Chemical Analyses in Diets, Feces, and Fish Fillets

The apparent digestibility of the dry matter, protein, fat, phosphorus, and energy were determined using yttrium content as a marker in feces and calculated as follows:

*ADC (apparent digestibility coefficient) = [100 − (marker in diet/marker in feces) × (% component in feces/% component in diet)] × 100*

Moisture, protein, fat, and ash contents in the fish fillet and diets were determined according to previously described procedures [31,32]. The moisture was calculated by drying samples at 105 °C for 24 h until constant weight. Protein contents were analyzed with the Kjeldahl method (N × 6.25), fat with dichloromethane extraction (Soxhlet), and ash content by heating the residue from the moisture determination in a muffle furnace at 550 °C for 24 h. Crude fiber content was calculated in the defatted fractions after digestion with sulfuric acid and sodium hydroxide and burning at 550 °C for 3 h. The starch (as nitrogen-free extract) was calculated according to [33]. All parameters were expressed as percentage in relation to dry matter. Analyses were conducted in quadruplicates.

### 2.6. Digestive Enzymes Activity

The digestive tracts of each rainbow trout specimen were dissected under a plastic platform directly placed on ice (at approx. 4 °C) to avoid tissue degradation and enzyme activation. Intestines were excised and homogenized (Ultra-Turrax T25 basic, IKA©—Werke) in cold Mannitol 50 mM and Tris–HCl 2 mM buffer, pH 7.0; while intestinal brush border membranes were purified according to the method described in [34] and stored at −80 °C prior to enzyme activity analysis. The specific activity of chymotrypsin, amylase, and aminopeptidase N were quantified. Chymotrypsin (E.C. 3.4.21.1) activity was assessed according to [35] using SAAPNA (N-succinyl-ala-ala-pro-phe p-nitroanilide) at 0.1 mmol L^−1^ in a Tris–HCl buffer (100 mmol L^−1^ + CaCl 210 mmol L^−1^ pH 7.8) as the substrate. Amylase (E.C. 3.2.1.1) activity was measured according to [36] using soluble starch (0.3%) dissolved in Na_2_HPO_4_ buffer (pH 7.4) as the substrate. Aminopeptidase N (E.C.3.4.11.2) was determined at 25 °C according to [37] using sodium phosphate buffer 80 mM (pH = 7.0) and L-leucine p-nitroanilide as the substrate (in 0.1 mM dimethyl sulfoxide). Enzymatic activities were normalized with the soluble protein content of crude enzyme extracts and quantified by means of the Bradford’s method [38] using bovine serum albumin as the standard. All the absorbances were measured in three technical replicates from each fish using a microplate reader (ELx800TM; BioTek Instruments, Inc., Winooski, VT, USA).

### 2.7. Histopathological Analysis

At the end of the experiment, the dissected proximal and distal intestine tissues of the rainbow trout were fixed via immersion in 4% buffered paraformaldehyde (pH 7.4) for 24 h at room temperature. Dehydration of fixed samples was performed by transferring samples in a sequential series of graded alcohol solutions (25%, 50%, 75%, and 100%). Samples were embedded in paraffin blocks, sectioned (3–5 µm), and stained with hematoxylin–eosin and Alcian blue (AB, pH = 2.5) periodic acid–Schiff (PAS) solutions to characterize the intestine histomorphology as well as to identify and quantify the goblet cell density in intestinal sections. All procedures were performed as described in [39,40,41].

Mounted sections were photographed with an Olympus EP50 camera coupled to an Olympus CX31 microscope, while image analysis was performed with Image J software. The height and number of fusions of villi; integrity of the brush border membrane; supranuclear vacuolization degree; localization of the nuclei in the enterocytes as well as the enterocytes’ height; submucosa, muscularia, and serosa layer width; and density of goblet cells were assessed at the proximal and distal intestine to determine the nutritional impact on the digestive system [42,43]. In particular, the brush border membrane was assessed according to the following scores: 1: 100% of the brush border membrane was fully preserved; 2: 80% of the brush border membrane was preserved; 3: 60% of the brush border membrane was preserved; 4: 40% of the brush border membrane was preserved; 5: only 20% of the brush border membrane was preserved; and 6: the brush border membrane was fully degraded. Regarding the supranuclear vacuolization degree, the following scores were considered: 1: 100% of the tips of the villi were filled with supranuclear vacuoles; 2: 75% of the tips of the villi were filled with supranuclear vacuoles; 3: 50% of the tips of the villi were filled with supranuclear vacuoles; 5: 25% of the tips of the villi were filled with supranuclear vacuoles; and 6: none of the tips of the villi were filled with supranuclear vacuoles. The localization of nucleus in the enterocytes was scored as: 1: nuclei of enterocytes were at the basal position; 2: nuclei were in an intermediate position; and 3: nuclei were in an apical position. The cellular impact of the total FM replacement on the digestive system was explored in 3 fish per experimental tank with 3 measurements/observations per section.

### 2.8. Circulating Non-Coding RNAs Analysis

#### 2.8.1. Isolation, Library Preparation, and Sequencing

Small non-coding RNAs (sncRNAs) were isolated from 150 μL of blood plasma samples using the miRNeasy Serum/Plasma Kit (Qiagen, Germany) following the manufacturer’s instructions, and assessment of the RNA quality and quantity was performed in a 2200 TapeStation Nucleic Acid system using High Sensitivity RNA ScreenTapes (Agilent, Santa Clara, CA, USA).

Library preparation of 10 samples (5 from fish fed with the FM diet and 5 from fish fed with the VM diet) was performed using 50 ng of sncRNA with the SMARTer smRNA-Seq Kit (Takara^®^) for Illumina, following the manufacturer’s protocol. The library size, purity, and concentration were evaluated with a 2100 Bioanalyzer (Agilent technologies). Normalized libraries were pooled and sequenced (single-end, 51 cycles) on a HiSeq2500 (Illumina, San Diego, CA, USA) platform using HiSeq2500 Rapid Run Sequencing (50SE, 1 lane). All sequencing data were submitted to the NCBI SRA database (accession number: PRJNA842865).

#### 2.8.2. Bioinformatic Analysis

Raw reads were adapter-trimmed using Cutadapt 2.8 [44] and quality-checked with FastQC v0.11.7 (>95% of base above Q30). Non-adapter, short (>17 bp after adapter trimming), and low-quality (with one or more N base) reads were removed. For each sample, the final processed reads were annotated using BLAST with rainbow trout, Atlantic salmon, and zebrafish (*Danio rerio*) miRs from miRBase v22.1 [45] and Cardona et al. [28] as well as a non-coding RNA database [46] to classify known miRs and other types of sncRNAs (e.g., tRNA, snRNA, snoRNA, etc.). Additionally, known/novel rainbow trout miRs were predicted using the recently assembled genome of rainbow trout (USDA_OmykA_1.1 assembly; GCA_013265735.3 [47]) with miRDeep2 [48]. Genome mapping and quantification were processed with Bowtie (v1.1.2) and STAR (v2.6.0c [49]) using RSEM (v1.3.1 [50]). Subsequently, differentially expressed (DE) miRs were identified with DESeq [51] while considering only DE miRs with an absolute log2fold change > 1 and a *q*-value < 0.05.

Risk of a false discovery of DE miRs due to hemolysis was initially checked by assessing oxyhemoglobin absorbance in the blood plasma samples (100 uL) at λ = 414 nm in a plate reader (Biotek, Gene 5 Microplate Reader, Winooski, VT, USA) and confirmed afterward with the in silico calculated ratio of miR-451 to miR-23a for each sample according to Blondal et al. [52].

#### 2.8.3. mRNA Target Prediction

The recently assembled transcriptome of rainbow trout [47] was used to predict mRNAs targeted by DE miRs. To explore potential targeted mRNAs, the corresponding 5′ and 3′ UTR regions and the coding sequence (CDS) were considered. Potential mRNA binding sites for miRs were identified using RNAhybrid [53]. An energy threshold of ≤−26 kcal mol^−1^ and a strict seed matching (no G:U allowed) in 2–8 nucleotides (nt) from the miR 5′ end was applied. The seeding region length was considered based on previous studies on miRs [54,55]. Gene Ontology (GO), overrepresentation (Fisher’s exact test with false discovery rate correction), and pathway analysis of predicted mRNAs were conducted using the Panther (http://www.pantherdb.org/; accessed on 18 April 2022) and Kyoto Encyclopedia of Genes and Genomes (KEGG; http://www.genome.jp/kegg/; accessed on 18 April 2022) platforms.

### 2.9. Statistical Analysis

Results are given as mean values ± standard deviations. All data were previously checked for normality (Kolmogorov–Smirnov test) and homoscedasticity of variance (Bartlett’s test). Unless otherwise indicated, the results were compared by means of a Student’s *t*-test to detect differences among the experimental groups using GraphPad Prism 5.0 (GraphPad Software, Inc., San Diego, CA, USA).

## 3. Results

### 3.1. Fish Growth Performance, Apparent Digestibility, and Fillet Composition

After 9 weeks of the feeding trial, the vegetable meal (VM) diet did not have a great impact on fish performance, nutrient apparent digestibility, or proximate composition of the fish fillet (Table 2). No significant differences were found between fish fed the FM and VM diets regarding fish growth in terms of the size and wet weight, condition factor, weight gain, specific growth rate (SGR), or feed conversion rate (FCR). In both experimental groups, the SGR and FCR mean values were good (around 3% and 0.77–0.79, respectively). Additionally, no differences were detected in the hepatosomatic index (HSI) between both groups, although the VSI was significantly higher in fish fed the VM diet than in fish fed the FM diet (18.13 ± 0.95 versus 13.84 ± 0.61, respectively; Student’s *t*-test: *p* < 0.05).

Similar to the impact on fish growth performance, no significant differences were identified between the FM and VM groups regarding the apparent digestibility of protein, phosphorus, and energy, although the apparent digestibility of fat was found to be significantly lower in fish fed the VM diet than in fish fed the FM diet (96.56 ± 0.29 versus 98.03 ± 0.48%, respectively; Student’s *t*-test: *p* < 0.05). Nevertheless, and although fat content in the fish fillets tended to be lower in fish fed the VM diet than in fish fed the FM diet (8.03 ± 0.23 versus 8.20 ± 0.65%, respectively), this difference in fat digestibility was not translated into a significantly different proximate composition of the fish fillets from both experimental groups (Student’s *t*-test: *p* > 0.05).

### 3.2. Digestive Enzyme Activities

The specific activities of chymotrypsin, aminopeptidase N, and α-amylase enzymes are presented in Table 3. While no significant differences were reported for chymotrypsin, the activity of aminopeptidase N was significantly higher in fish fed the VM diet than in fish fed the FM diet (32.02 ± 2.28 versus 21.60 ± 4.07 U/mg, respectively; Student’s *t*-test: *p* < 0.05). The activity of α-amylase was also affected by the diet, but in this case, it was significantly lower in fish fed the VM diet (30.81 ± 8.07 versus 51.72 ± 9.40 U/mg, respectively; Student’s *t*-test: *p* < 0.05).

### 3.3. Histopathological Analysis of Proximal and Distal Intestines

**Figure 1:**
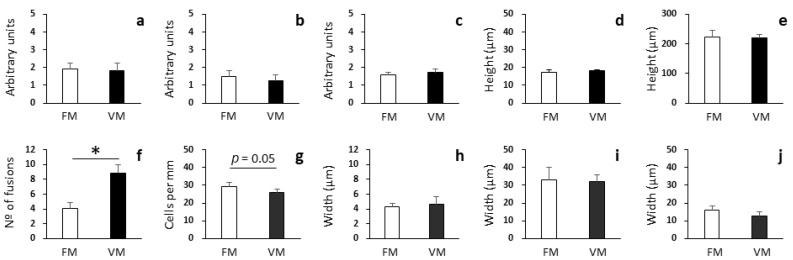
Histological analysis of proximal intestine of rainbow trout fed with fish meal (FM) and vegetable meal (VM) diets. Brush border integrity (**a**), degree of supranuclear vacuolization in enterocytes (**b**), enterocyte nuclei position (**c**), enterocyte height (**d**), height of villi (**e**), number of villi fusions per histological section (**f**), goblet cell density (**g**), width of submucosa layer (**h**), width of muscular layer (**i**), and width of serosa layer (**j**). Arbitrary units for brush border integrity (**a**), degree of supranuclear vacuolization in enterocytes (**b**), and enterocyte nuclei position (**c**) are according to scores defined in the Materials and Methods section. Asterisks denote significant differences among experimental groups (Student’s *t*-test: *p* < 0.05; n = 4). Of note, among the different parameters evaluated, only the number of fusions was found to be significantly different between the two experimental groups, while the goblet cell density was close to be significant (Student’s *t*-test: *p* = 0.05).

**Figure 2:**
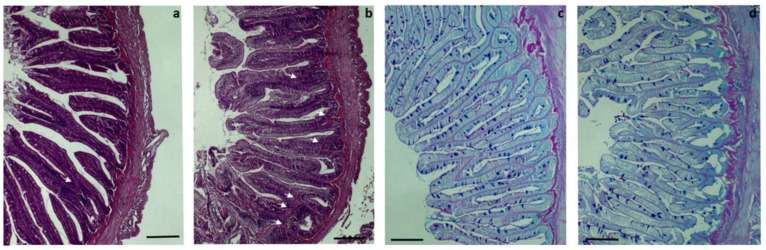
Histopathology of proximal intestine of rainbow trout fed with fish meal (FM) and vegetable meal (VM) diets. Examples of a low (FM) (**a**) and high (VM) (**b**) number of villi fusions (white arrows) and examples of a high (FM) (**c**) and low (VM) (**d**) density of goblet cells per section of proximal intestine. Note also the higher width of mucosal folds in fish fed the VM diet (**b**) than in those fed the FM diet (**a**). Scale bar: 100 µm.

In the proximal intestine, among the different parameters evaluated to elucidate the potential impact of full FM replacement, significant differences were only found regarding the number of villi fusions per section (Figure 1). In fish fed the VM diet, a higher number of fusions (8.83 ± 1.14) were recorded than in fish fed the FM diet (4.08 ± 0.74; Figure 2a,b; Student’s *t*-test: *p* < 0.05). Also, an increased width of villi in fish fed the VM diet was evidenced (Figure 2a,b). However, no significant differences were found in the brush border integrity; degree of supranuclear vacuolization in enterocytes; enterocyte nuclei position; enterocyte height; height of villi; width of the submucosa, muscular, and serosa layers or goblet cell density, although the reduction in the goblet cell density in fish fed VM (from 29.36 ± 2.08 to 25.75 ± 2.19) was almost significant (Figure 2c,d; Student’s *t*-test: *p* = 0.054).

**Figure 3:**
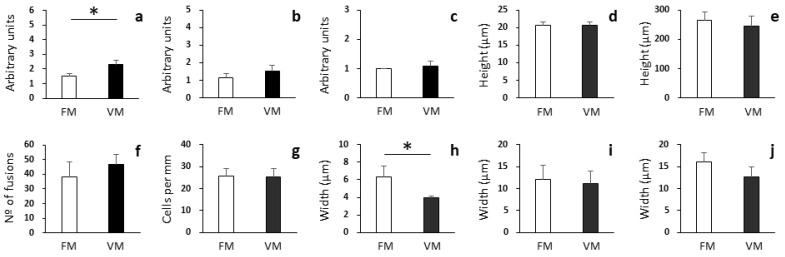
Histological analysis of distal intestine of rainbow trout fed with fish meal (FM) and vegetable meal (VM) diets. Brush border integrity (**a**), degree of supranuclear vacuolization in enterocytes (**b**), enterocyte nuclei position (**c**), enterocyte height (**d**), height of villi (**e**), number of villi fusions per histological section (**f**), goblet cell density (**g**), width of submucosa layer (**h**), width of muscular layer (**i**), and width of serosa layer (**j**). Arbitrary units for brush border integrity (**a**), degree of supranuclear vacuolization in enterocytes (**b**), and enterocyte nuclei position (**c**) are according to scores defined in the Materials and Methods section. Asterisks denote significant differences among experimental groups (Student’s *t*-test: *p* < 0.05; n = 4). Of note, among the different parameters evaluated, only the brush border integrity and the width of submucosa layer were found to be significantly different between the two experimental groups.

**Figure 4:**
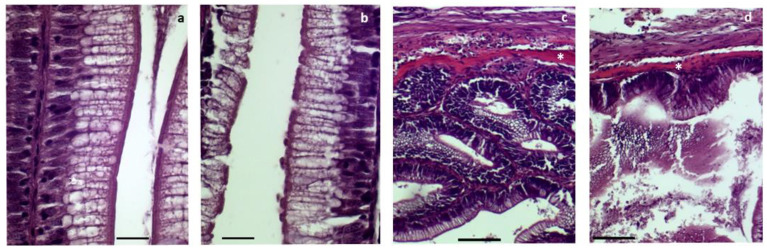
Histopathology of distal intestine of rainbow trout fed with fish meal (FM) and vegetable meal (VM) diets. Examples of brush border integrity fully preserved (FM) (**a**) or partially degraded (VM) (**b**) and examples of wide (FM) (**c**) and narrow (VM) (**d**) submucosa layers (asterisks). Scale bar: 10 µm in (**a**,**b**); 50 µm in (**c**,**d**).

In the distal intestine, significant differences were found regarding the brush border integrity and the width of the submucosa layer (Figure 3; Student’s *t*-test: *p* < 0.05). A lower brush border integrity was observed in fish fed the VM diet (2.33 ± 0.27 arbitrary units) than in fish fed the FM diet (1.50 ± 0.19 arbitrary units; Figure 4a,b). Similarly, a lower width of the submucosa layer was more associated with fish fed the VM diet (3.98 ± 0.17 µm) than fish fed the FM diet (6.35 ± 1.18 µm; Figure 4c,d).

### 3.4. PCA of Growth Performance, Proximal Composition, Enzyme Activities, and Histopathological Parameters

**Figure 5:**
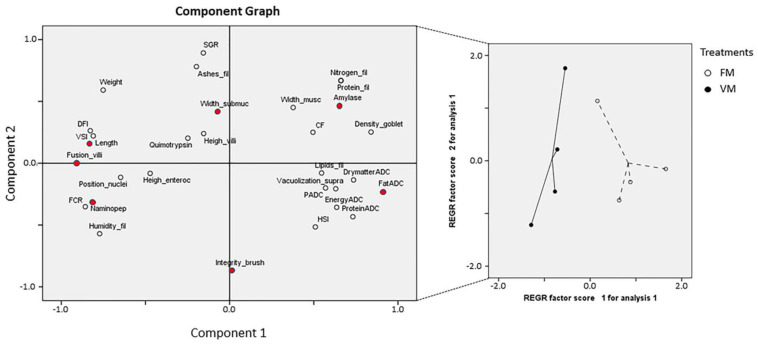
Scatter plot of experimental groups (with centroid distribution of the replicates) separated by the two principal components obtained from a principal component analysis (PCA) to reduce the dimensionality of the dataset when rainbow trout specimens were fed experimental diets (FM and VM). Red circles denote parameters reported to be statistically different between fish fed with FM and VM diets. Components 1 and 2 explained 59.56% of the variance between both experimental groups.

Although no extensive differences were found in the diverse set of parameters evaluated between both experimental groups, a PCA of the recorded parameters showed that the FM and VM replicates were clustered separately by the two principal components, explaining 59.56 % of the variance encountered between both experimental groups (Figure 5; Supplementary Table S1).

### 3.5. Circulating Non-Coding RNAs

At least three fish from each replicate were sampled for blood plasma. Among them, the five with the lower risk of hemolysis and lower absorbance of oxyhemoglobin at λ = 414 nm (ranging from 0.20 to 0.34; see Supplementary Table S2) were selected and sequenced.

The global small RNA-Seq output is compiled in Table 4. About 15 to 22 million raw reads were obtained from each sample, resulting in 6 to 12 million trimmed reads (40–64%) with a consistent GC content and a good quality score (Q30) in 95% of the reads. Small RNA-Seq analysis showed that 40,643 to 183,007 reads were annotated as circulating miRs of rainbow trout, representing 0.60 to 1.58% of the total reads. All these annotated reads corresponded to a total of 497 mature miRs (Supplementary Table S3).

**Figure 6:**
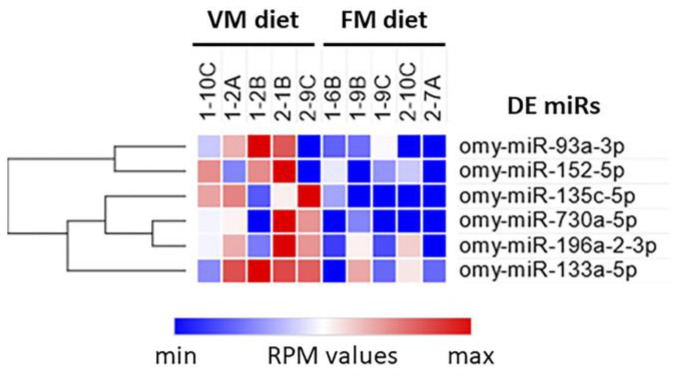
Heatmap representation of differentially expressed circulating miRs in blood plasma from rainbow trout fed a vegetable meal (VM) diet versus a fish meal (FM) diet. The heatmap was built with Morpheus (https://software.broadinstitute.org/morpheus; accessed on 10 October 2022).

A comparative analysis of samples from rainbow trout specimens fed with FM or VM diets identified six differentially expressed (DE) miRs (Table 5; Figure 6). All of them were more highly expressed in blood plasma from fish fed the VM diet than in fish from the FM group. While the Log2(Fold change) of omy-miR-730a-5p and omy-miR-135c-5p were above 3, a more modest Log2(Fold change) (between 1 and 2) was observed for omy-miR-93a-3p, omy-miR-152-5p, omy-miR-133a-5p, and omy-miR-196a-3p. In order to expand our understanding of how a diet devoid of FM impacts fish physiology, potential mRNAs targets of DE miRs were in silico identified.

**Figure 7:**
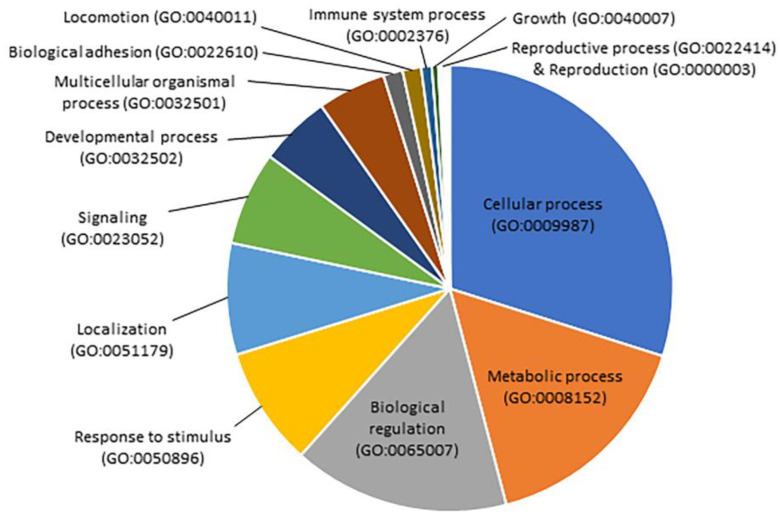
Pie chart of GO biological process of predicted mRNAs targeted by differentially expressed (DE) miRs from blood plasma of rainbow trout fed vegetable meal (VM) and fish meal (FM) diets. Note that the mRNAs were predicted to be targeted by miRs upregulated in blood plasma from fish fed VM diets when compared to those fed FM diet. GO analysis of predicted mRNAs was conducted with the transcriptome of rainbow trout (USDA_OmykA_1.1 assembly; GCA_013265735.3; [47]) and bioinformatic platforms Panther (http://www.pantherdb.org/; accessed on 18 April 2022) and Kyoto Encyclopedia of Genes and Genomes (KEGG; http://www.genome.jp/kegg/; accessed on 18 April 2022).

A total of 3770 unique protein-coding genes were bioinformatically predicted as being targets of these six DE miRs (Supplementary Table S4). A Gene Ontology (GO) analysis of the functionally well-characterized mRNAs (795 in total) showed that they were involved in 17 different biological processes (Figure 7). Cellular, metabolic, biological regulation, and response to stimulus were the biological processes involving more hypothetically targeted genes. Among the genes involved in metabolic processes, several predicted targets were specifically involved in fatty acid metabolism (*long-chain fatty acid transport protein 1*, *fatty acid binding protein 1*, *fatty acid synthase*, *polyunsaturated fatty acid 5-lipoxygensase*, *long-chain-fatty-acid-CoA ligase 3* and *4*, and *long-chain-fatty-acid-CoA ligase ACSBG2*).

An overrepresentation analysis identified 32 different GO biological process (Table 6). Among them, the major GO biological processes were telomere maintenance, morphogenesis of an epithelium, plasma membrane bounded cell projection organization, intracellular transport, cell development, nervous system development, animal organ development, and negative regulation of the apoptosis process; all of them were enriched except negative regulation of the apoptosis process. Telomere maintenance and telomere organization were the most highly enriched GOs (+6.93 and +6.77 folds), including the same nine genes (*atr*, *tinf2*, *ctc1*, *xrcc6*, *atm*, *prkdc*, *hnrnpua*, *dclre1a,* and *zgc:113263*). Only six protein classes were found to be overrepresented: the microtubule binding motor protein, microtubule or microtubule-binding cytoskeletal protein, ATP-binding cassette (ABC) transporter, membrane traffic protein, immunoglobulin receptor superfamily, and defense/immunity protein; the last two were less represented (Table 7). Regarding the protein class that was most highly enriched, the microtubule binding motor protein (PC00156; +5.62 folds enrichment) exclusively included *kinesin-related* genes (*kif1b*, *kif3a*, *kif3c*, *kif5c*, *kif14*, *kif17*, *kif23*, and *kifap3a*) and *dyneins* (*danh5*, *danh5l*, and *danh12*). The ATP-binding cassette (ABC) transporter (PC00003), which was the second most highly enriched protein class (+5.49 fold enrichment), included nine genes (*abca1b*, *abca12*, *abcb6a*, *abcb9*, *abcb11a*, *abcc2*, *abcc3*, *abcg2b*, and *abcg4a*), three of which are involved in the bile secretion KEGG pathway (Supplementary Figure S1).

## 4. Discussion

### 4.1. FM Replacement with High SBM Content Impacts Rainbow Trout Physiology

Total FM replacement with SBM in a high % (>20%) is known to induce lower survival and reduced growth and can have a deep physiological impact on salmonids or high-trophic-level (“carnivorous”) fish [56]. In this study, total FM replacement (including a high (~30%) SBM content) evidenced a fish physiology impairment in terms of the apparent fat digestibility, viscerosomatic index (VSI), digestive enzyme activity, and some histopathological alterations in the intestine after a 9-week period. However, this was not translated to reduced rainbow trout growth. Contradictory results on rainbow trout growth have been reported when the fish were fed diets with high SBM inclusion. While some recent research works reported a lower fish growth when using high dietary SBM content [57,58], Refstie et al. [59] did not find significant differences. Discrepancies in fish growth might be related to the control and experimental feed formulations, length of the feeding period, and/or strains used. For instance, Blaufuss et al. [57] compared a control diet containing 30% FM and 0% SBM and an experimental one with 0% FM and 40% SBM during a 12-week period. Vélez-Calabria et al. [58] compared fish growth using diets including 10 or 20% of FM and 30 or 20% of SBM (respectively) with a diet depleted of FM but containing 40% of SBM during 11 weeks. In contrast, Refstie et al. [59] compared two experimental diets containing 50 and 32% of FM and 0 and 30% of SBM, respectively; while low (12%) or no FM inclusion and 8 and 26% of SBM was considered in our control (FM) and experimental (VM) diets, respectively. 

Despite of the lack of growth reduction in fish fed the VM diet, a lower apparent fat digestibility and α-amylase activity, an increased aminopeptidase N activity and VSI, and histopathological alterations in the proximal and distal intestines clearly evidenced a physiological impact on rainbow trout, which was consistent with previous research works. A lower apparent fat digestibility was also observed when the SBM content was increased in rainbow trout diets [57,59]. This physiological impact was intimately associated with reduced lipid digestion and absorption of lipids as evidenced by the altered transcriptome and miRnome in studies using a diverse set of fish species [13,14,15,22,23] and discussed later. Different pancreatic digestive enzymes are essential for the particular digestion of the nutrients provided by the diet: trypsin, chymotrypsin, and aminopeptidase N for proteins; lipase for lipids; and amylase for carbohydrates [9]. It has long been demonstrated that particular SBM components (antinutritional factors) can reduce digestive enzymes’ activity [10,11]. While no differences in chymotrypsin activity were reported in the present study, total FM replacement in the VM diet (with increased SBM and wheat meal dietary content) induced higher aminopeptidase N and lower α-amylase activities. Higher activities of trypsin, chymotrypsin, elastase, and lipase in Atlantic salmon fed with increasing dietary levels of SBM were reported in a short-term study (up to a 7-day feeding period) [60]. On the other hand, red sea bream (*Pagrus major*) fed SBM diets showed lower content and activity of pancreatic digestive enzymes when compared to fish fed FM [61]. Similarly, gilthead seabream (*Sparus aurata*) larvae fed on diets containing SBM had significantly reduced trypsin, chymotrypsin, and amylase activities [62]. Aminopeptidase N activity was not significantly affected by the replacement of FM with other vegetable protein resources in two fish species (gilthead sea bream and goldfish (*Carasius auratus* [63]). The higher aminopeptidase N activity reported here might be physiologically relevant because this enzyme is in charge of the final digestion of peptides derived from the protein hydrolysis performed by gastric and pancreatic proteases [64]. FM replacement with other plant-protein sources did not reduce α-amylase activity in rainbow trout [65], but high dietary SBM inclusion (concomitant with a lower starch or wheat meal content) decreased α-amylase activity in different fish species [66,67], which was in line with the present results. This reinforces the hypothesis that high SBM content might be the main driver of lower amylase activity in rainbow trout fed a VM diet rather than the wheat meal content. In the present study, as well in the literature, a higher VSI was reported in different fish species when FM was replaced with high SBM content [68]. This was consistent with the higher number of villi fusions and the increased width of mucosal folds in fish fed with a high SBM content diet (VM) and in line with the most characteristic outcome of FM replacement with high SBM content (among other vegetable protein sources) in salmonids and high-trophic-level (“carnivorous”) fish: the induction of intestinal inflammation (enteritis). This response depends on the SBM content, whether the SBM was processed (fermented, cooked, and/or enzymatically pretreated), the fish species, the strain and size considered, the feed formulation, and the length of the feeding period [57,60,69,70,71]. This effect on the intestine seems to be due to different saponin fractions present in SBM, but other SBM compounds also may contribute to this intestinal inflammation [42,56,72]. Different histopathological parameters (e.g., the height of mucosal folds and enterocytes, the width of mucosal folds, lamina propria and/or submucosa, muscular and serosa layers, brush border integrity, supranuclear vacuolization, and leukocyte infiltration, among others) are commonly used to monitor the health of the fish intestine and the impact of SBM dietary inclusion [15,42]. All of these histopathological features related to high dietary SBM content have been previously reported in different fish species, including rainbow trout [9,15,56,68,71,73,74]. Here, a high dietary SBM content did not reduce the height of mucosal folds and enterocytes or the supranuclear vacuolization. Nevertheless, an increased number of villi fusions, a higher width of mucosal folds, and a reduced brush border integrity and width of the submucosa layer was observed in rainbow trout fed with the VM diet, which was in line with previous research studies showing an increased number of villi fusions [71,73], a higher width of mucosal folds due to the widening of lamina propria [71,73], and/or a lower brush border integrity [9,74]. These smaller effects of increased dietary SBM content in the intestinal tissue of rainbow trout were more consistent with the lower SBM inclusion in our VM diet than those tested in other reports (e.g., [15]) and with the fact that rainbow trout are usually less affected than other species (even when considering freshwater fish species such as Atlantic salmon [59]). Interestingly, considering the role of goblet cells on immunity [75], although not statistically significant, the reduced goblet cell density according to the level of dietary SBM inclusion reported here might have some implications for rainbow trout immunocompetence. Furthermore, although a lower impact than previously described was observed when comparing the growth performance and physiology in rainbow trout fed FM and VM diets, both groups were clustered separately when the evaluated parameters were considered globally (PCA), evidencing that both groups were under a different physiological condition.

### 4.2. The Expression of Particular Circulating miRs from Blood Plasma Is Associated with High Dietary SBM Content

Mature miRs are transcripts that are 18–24 nt in length that do not code for proteins. MiRs play a key role in post-transcriptional gene regulation (normally repressing gene translation, but in some cases gene transcription or translation activation also were reported [76]) and showed very interesting features for their use as integrative biomarkers in different diseases and disorders [18,77]. They are more stable in response to environmental conditions than mRNAs, are actively released from cells to blood plasma, and sampling circulating miRs from blood plasma does not require animal euthanasia [18]. Indeed, their presence in blood plasma is now considered as another system of cell–cell communication [24], and very recent research works supported their use for monitoring nutritional, health, and reproductive status in different fish species (reviewed in [18]).

Between 236 and 318 different mature miRs were identified and quantified in the blood plasma of the sampled fish, and a total of 497 different mature miRs were identified. Previous works reported a similar range (196–500) of mature circulating miRs in blood plasma from different fish species [25,26,27,28,78]. Here, among the miR populations identified in the blood plasma, omy-miR-730a-5p, omy-miR-135c-5p, omy-miR-93a-3p, omy-miR-152-5p, omy-miR-133a-5p, and omy-miR-196a-3p were found to be more highly expressed in fish fed with the VM diet than in fish fed with the FM diet. Except for miR-196a-3p, all DE circulating miRs were also found in rainbow trout blood plasma by Cardona and colleagues [28]. Other nutritional interventions in fish species showed how some miRs and/or piwi-interacting RNAs (piRNAs) were particularly related to cholesterol synthesis and efflux or glucose phosphorylation [79], dietary vitamin K content [25], FM and FO replacement by plant sources [80], and/or different feeding regimes (*ad libitum* vs. food restriction [28]). Thus, the results from the present study increase our understanding of the biological pathways altered under high dietary SBM content.

Little is known about the particular biological processes and related pathways in which these DE miRS are involved. The expression of miR-730a-5p was detected in the mature testis of Atlantic salmon [81], and was downregulated in the liver when genetically improved farmed tilapia (*Oreochromis niloticus*) was submitted to a heat stress (from 28 to 37.5 °C [82]). It was found that miR-152-5p seems to act as a tumor suppressor in different sorts of cancers and/or controlling fibrosis progression [83,84,85]. Similarly, miR-133a-5p overexpression significantly suppressed the viability of particular prostate cancer cell lines [86], while miR-93a-3p was downregulated in blood plasma from Atlantic salmon infected with infectious salmon anemia virus (ISAV [27]). Among the six DE circulating miRs reported here, three were found to be associated with lipid metabolism. Although miR-135c-5p was found to be mainly expressed in the brain [87], a lower expression in liver tissue from Atlantic salmon families selected for high *delta 6 desaturase isomer b* gene expression was reported [20]. In addition, miR-196a has been associated with brown adipogenesis [88], bone mineral density [89], and ulcerative colitis [90]; while miR-133a regulates adipocyte browning [91]. Although there is no evidence of brown or beige adipocytes in teleost fish [92], higher expression was found in blood plasma from specimens fed with the VM diet, showing a lower apparent fat digestibility. Moreover, miR-133a-2-5p was also upregulated in the intestine of juvenile grass carp (*Ctenopharyngodon idella*) when fed with 40% SBM [93], which was in line with the present results.

Although using the same fish species (rainbow trout) and similar experimental setup (FM replacement with plant sources), Zhu and co-workers [79,80] reported miR-33a, miR-122, and miR-128 to be DE through a qPCR approach, while these miRs were not significantly DE in our study. While Zhu et al. [79,80] explored the differences between trout fed a diet based on FM (58.4%) and FO (14.1%) and those fed a diet totally depleted of FM and FO, we compared miRs from blood plasma of trout fed with a low FM content (12%) and low FO (6.7%) with those fed a diet devoid of FM and low FO (7%) and particularly rich in SBM. While the FM diet used here was more representative of the current feed’s formulation for rainbow trout, a smaller difference (relative to the control) in the feed’s formulation might explain the discrepancies encountered regarding the reported DE miRs. Indeed, DE miRs reported by Zhu et al. [79,80] were found to be related to hypocholesterolemia, potentially due to FO being totally depleted in their experimental diets (a condition not represented in our experimental diets).

Previous genomic studies conducted at the tissue level identified several GOs commonly altered in the liver and/or intestine when fish species were fed with high SBM content and/or high FM replacement with plant protein sources, including lipid (fatty acid) metabolism, ion transport, bile acid biosynthesis and secretion, steroid biosynthesis, cell proliferation and apoptosis, oxidative stress, digestion and absorption of nutrients (proteins, lipid, vitamins, and minerals), purine metabolism, and immune system response [13,14,15,23,73,93,94]. Although tissue mRNA expression was not explored here, bioinformatic prediction of potential mRNAs targets of DE miRs can help to understand how DE miRs might impact particular physiological processes [18] and—in the present case—to provide new insights on the limiting factors of high SBM inclusion in aquafeeds.

Among the mRNAs bioinformatically predicted to be targeted by the DE miRs of the present study, the GO analysis of the 795 functionally characterized mRNAs identified cellular, metabolic, biological regulation, and response to stimuli as the most highly represented biological processes. Within the targeted genes involved in metabolic processes, several were related to fatty acid metabolism and trafficking (*long-chain fatty acid transport protein 1*, *fatty acid binding protein 1*, *fatty acid synthase*, *polyunsaturated fatty acid 5-lipoxygensase*, *long-chain-fatty-acid-CoA ligase 3* and *4*, and *long-chain-fatty-acid-CoA ligase acsbg2*). The expression of some of these genes was also found to be altered when Atlantic salmon were fed on a diet with a high FM replacement with plant proteins sources, and particularly during soybean-meal-induced enteritis (*e.g.*, *acyl-CoA synthetase long-chain family member 5* and *6* downregulation [15]). Indeed, some of these predicted genes are specifically involved in fish adipogenesis [92]. Furthermore, an overrepresentation analysis of the biological processes provided a new, wider, and specific insight on the pathways regulated by these DE miRs. Transport (GO:0006810) was in agreement with the altered transport reported at transcriptional level in previous genomic studies in fish fed with diets including a high SBM content and/or where FM and FO were totally replaced with plant sources. Several studies reported the altered expression of genes from different solute carrier families (e.g., *slc26a6l* and *slc6a19* downregulation), ATP-binding cassette (e.g., *abca1* upregulation), and sodium-associated and/or choline transporter genes [21,73,94]. Likewise, members of the *solute carrier family 22*, *choline transporter-like protein 4*, *choline O-acetyltransferase*, or *ATP-binding cassette transporter A1* (*abca1*), among others, were predicted to be targets of the identified DE miRs. Another biological process overrepresented by mRNAs targeted by DE miRs was morphogenesis of an epithelium (GO:0002009), likely associated with the lower brush border integrity exclusively observed at the distal intestine in rainbow trout fed high dietary SBM content (present study) and in line with the distal intestine being recognized as the site where the SBM has its greatest inflammatory impact; as a region densely filled with associated immune cells and where the local immune response is usually higher [58]. Such impact has been related to pathways directly linked to the intestinal barrier function and permeability (e.g., remodeling of epithelial adherent junctions and epithelial adherent junction signaling) in Atlantic salmon when fed a high SBM content diet [15] or different tight junction proteins such zonula occludens-1 or myosin light chain kinase that maintain the intestinal integrity [9]. Genes potentially targeted by DE miRs related to brush border integrity and morphogenesis of an epithelium such as *zona occludens-1* and *3*, several *laminins*, *sialomucins,* and *mucins* (including *2-like*, *5B*, *5B-like,* and *5AC* isoforms of the last gene) were identified. Previous genomic approaches also identified some laminins (*laminin beta 4* was downregulated [14]) and mucins (*mucin 5ac* was upregulated but *mucin 5d* was downregulated [94]) as DE genes under high dietary SBM content. Unexpectedly, telomere maintenance (GO:0000723) and organization (GO:0032200) were overrepresented. Activation of telomere maintenance mechanisms is required to prevent genome instability and to establish cellular immortality [95]. Moreover, epithelial integrity disruption in the human gastrointestinal tract by telomere dysfunction has been associated with gastrointestinal diseases [96] and particularly with intestinal inflammation [97]. Therefore, our results unveiled another new potential gene network responsible for the observed physiological impact of high SBM content in fish species.

The GO analysis of bioinformatically predicted mRNA targets also showed six overrepresented protein classes. Regarding the microtubule binding motor protein (PC00156) and microtubule or microtubule-binding cytoskeletal protein (PC00157), although largely known to be involved in maintaining cell structure and forming the cytoskeleton, recent studies suggest they are also important players in the innate and adaptive immune systems, particularly in the gut (reviewed in [98]). Indeed, disruption of the gut cytoskeleton is relevant to several systemic disorders such as bowel disease, and these protein classes might be another indication of the large impact of SBM dietary content on the fish immune system, which was one of the most commonly reported GOs altered by SBM and/or plant-based diets in fish species in previous genomic studies [14,15,23,73,93,94]. Also, both the immunoglobulin receptor superfamily (PC00124) and defense/immunity protein (PC00090) were the protein classes underrepresented by mRNA targets, suggesting that the targets of DE miRs were not dealing mainly with these specific immune pathways. In contrast, the ATP-binding cassette (ABC) transporter (PC00003) and membrane traffic protein (PC00150) were the two other protein classes overrepresented, further evidencing the potential role of the DE miRs on the altered transport of biological relevant compounds related to high dietary SBM content. Within the nine *ABC transporter* genes potentially targeted, the *abca1b* mammalian ortholog (*abca1*) is known to participate in fat digestion and absorption as well as in cholesterol synthesis, while orthologs of *abcb11a*, *abcc2*, *abcc3,* and *abcg2b* have been reported to control bile secretion [99,100,101,102,103]. ABC transporters are critical in metabolic diseases due to their capacity to transport lipids and participate in cholesterol uptake, biosynthesis, and storage to maintain cholesterol homeostasis [104]. Reduced content of cholesterol and bile acids in fish fed diets with high FM replacement with SBM was previously correlated with lower expression of cholesterol (*abcg5*) transporter in the distal intestine [13]. Indeed, a reduced hepatic production of bile was reported as an important factor of the enteritis induced by SBM in salmonids [13,60,105,106]. Bile is secreted into the proximal intestine, where it acts as a surfactant by emulsifying lipids into micelles, which enhances digestion by allowing for more cleavage sites for lipase. Some research studies already explored the addition of bile acids to mitigate the inhibited lipid digestion when feeds were based on protein and oil plant sources, including SBM (reviewed in [72]). Our bioinformatic results predicted that different mRNAs involved in bile secretion were targets of our DE miRs, consistent with the lower apparent fat digestibility. Until now, two causes of bile acid status disturbance in fish were considered: (i) increased excretion/decreased reabsorption; and/or (ii) decreased bile acid synthesis [72]. The present study points out that reduced bile secretion might be another cause. Since bile also improves the absorption of other fat-soluble nutrients (such as vitamins A, D, E, and K; carotenoids; and astaxanthin) [72], new nutritional requirements for these fat-soluble vitamins might be expected in new feed formulations based on alternative sources to FM and FO. Indeed, higher vitamin A (VA) requirements were suggested for rainbow trout when fed on a diet based on plant ingredients [107]. Some genes involved in the metabolism of some fat-soluble vitamins, and particularly of VA (*cytochrome P450 26A1*, *cytochrome P450 26A1-like*, *retinol dehydrogenase 12* and *10*, and *retinal dehydrogenase 1*), were also predicted as targets of the DE miRs.

Different methodologies have allowed researchers to expand our understanding of how miRNAs are involved in different biological processes. In the present study, bioinformatic prediction of mRNA targets was used to explore which particular biological processes might be affected in rainbow trout when fed with high dietary SBM content. Algorithm predictions are based on different parameters such as seed match and complementarity, conservation, free energy, and site accessibility [18]. Nevertheless, even when using the most accurate predictions, those including minimum free energy [53] and/or flanking AU nucleotide content [108] among other parameters, some false positives might also be predicted [18]. Although an evolutionary conservation of the binding site of the mRNA from different species might be considered, both miR and mRNA sequences can diverge even within teleost species [109]. Thus, while a functional validation of the miR–mRNA interaction might be needed, bioinformatic prediction provides a promising initial perspective of the molecular pathways controlled by DE miRs. Therefore, future research work is specifically needed to validate predicted miR–mRNA interactions and explore their physiological consequences (e.g., regarding lipid metabolism, immune system response, epithelial integrity disruption, and bile acid status).

## 5. Conclusions

Deeper understanding of the mechanisms and the availability of less invasive (non-lethal) monitoring tools to diagnose metabolic disorders may prevent the occurrence of intestinal diseases such as SBM-induced enteritis in farmed teleost fish. Here, the isolation and quantification of circulating miRs in blood plasma related to the physiological impact of total FM replacement with high dietary SBM content on rainbow trout was conducted for the first time. Six different miRs were found to be differentially expressed when comparing rainbow trout fed with a diet representative of current commercial formulations (low FM and FO content—FM diet) and a diet totally devoid of FM (replaced with SBM) and with a low FO content (VM diet). While bioinformatic prediction of target mRNAs identified biological processes already known to be altered (e.g., lipid metabolism, immune system response, epithelial integrity disruption, and bile acid status), more specific (e.g., bile secretion) and new related pathways (e.g., microtubule binding motor and cytoskeletal proteins as well as telomere maintenance and organization) were revealed. The expression levels of specific circulating ncRNAs in fish blood plasma, particularly miRs, have been directly associated with the physiological consequences of a high dietary SBM content, suggesting they might be suitable, integrative, and less invasive biomarkers to be screened for improving FM replacement in both experimental and farming conditions. In this sense, future research work is required to evaluate if their expression level is reproducible, sensitive, and reversible under this disorder (and/or other conditions) in order to be fully considered as reliable biomarkers in fish physiology.

## Figures and Tables

**Table 1:** Ingredients and proximate composition of experimental diets.

Ingredients (g/100 g, on Wet Basis)	FM	VM
Fishmeal Super Prime (Diamante)	12.00	0.00
Soy protein concentrate (Soycomil)	20.00	20.00
Wheat gluten	8.50	8.50
Corn gluten	5.00	5.00
Soybean meal 48	8.00	26.20
Rapeseed meal	5.00	5.00
Wheat meal	16.38	8.37
Fish oil	6.72	7.05
Rapeseed oil	14.56	15.28
Linseed oil	1.12	1.18
Vit & Min Premix INVIVO 1%	1.00	1.00
Antioxidant powder (Verdilox)	0.20	0.20
Sodium propionate	0.10	0.10
MAP (monoammonium phosphate)	0.70	1.40
L-lysine	0.20	0.20
DL-methionine	0.50	0.50
Yttrium oxide	0.02	0.02
**Total**	**100.00**	**100.00**
**Proximate Composition (Dry Matter Basis)**	**FM**	**VM**
Crude protein, % feed	38.00	38.00
Crude fat, % feed	25.00	25.00
Fiber, % feed	2.30	2.70
Starch, % feed	11.40	7.40
Ash, % feed	6.10	5.50
Gross energy, MJ/kg feed	23.40	23.40
Total P, % feed	0.80	0.80

**Table 2:** Growth performance, somatic indexes, apparent protein digestibility coefficients of diets, and fillet analysis in rainbow trout fed with fish meal (FM) and vegetable meal (VM) diets.

Days	Parameter	FM	VM
0	IBW (g)	23.24 ± 0.20	23.31 ± 0.22
	ITL (cm)	12.44 ± 0.10	12.51 ± 0.04
63	FBW (g)	148.84 ± 6.46	155.96 ± 3.41
	FTL (cm)	21.87 ± 0.35	22.34 ± 0.39
	CF	1.34 ± 0.02	1.30 ± 0.06
	WG (%)	540.39 ± 23.32	569.18 ± 16.94
	DFI (%)	2.06 ± 0.06	2.16 ± 0.02
	SGR (%/day)	2.95 ± 0.06	3.02 ± 0.04
	FCR	0.77 ± 0.01	0.79 ± 0.02
	HSI (%)	1.62 ± 0.11	1.49 ± 0.04
	VSI (%)	13.84 ± 0.61	18.13 ± 0.95 *
**Apparent digestibility coefficients (%)**			
Dry matter		82.94 ± 1.78	80.56 ± 0.97
Protein		93.70 ± 0.82	92.70 ± 0.67
Fat		98.03 ± 0.48	96.56 ± 0.29 *
Phosphorus		61.44 ± 3.34	58.50 ± 2.66
Energy		87.36 ± 1.19	86.03 ± 0.92
**Proximate analysis of muscle (% on wet weight basis)**			
Moisture		72.09 ± 0.05	73.16 ± 1.02
Protein		18.25 ± 0.32	17.29 ± 0.73
Fat		8.20 ± 0.65	8.03 ± 0.33
Ash		1.46 ± 0.07	1.52 ± 0.15

Values are expressed as mean ± standard deviation. Asterisks denote significant differences among experimental groups (Student’s *t*-test: *p* < 0.05; n = 4). IBW, initial body weight; ITL, initial total length; FBW, final body weight; FTL, final total length; CF, condition factor; WG, weight gain; DFI, daily feed intake; SGR, specific growth rate; FCR, feed conversion ratio; HSI, hepatosomatic index; VSI, viscerosomatic index. Values of VSI and fat apparent digestibility coefficients from the VM group (significantly different from the ones of the FM group) are shown in bold letters to highlight them.

**Table 3:** Specific activities of digestive enzymes in rainbow trout fed with fish meal (FM) and vegetable meal (VM) diets.

Enzyme	Units	FM	VM
Chymotrypsin	mU/µg	22.05 ± 8.29	23.32 ± 4.99
Aminopeptidase N	U/mg	21.60 ± 4.07	32.02 ± 2.28 *
α-amylase	U/mg	51.72 ± 9.40	30.81 ± 8.07 *

Values are expressed as mean ± standard deviation. Asterisks denote significant differences among experimental groups (Student’s *t*-test: *p* < 0.05; n = 4).

**Table 4:** Summary of the sequenced small non-coding RNAs in circulation in rainbow trout fed with fish meal (FM) and vegetable meal (VM) diets.

Experimental Group	Fish Code	Total Raw Reads	After Trimming		GC (%)	Q30 (%)	miRNAs	
			# of Reads	%			# of Reads	%
FM	1-6B	20,732,616	11,067,387	53.38	57.82	95.51	157,593	1.42
	1-9B	15,395,251	8,972,934	58.28	56.71	95.37	136,808	1.52
	1-9C	18,470,158	9,318,040	50.45	56.07	95.45	122,959	1.32
	2-7A	15,098,313	6,595,979	43.69	52.30	95.71	40,643	0.62
	2-10C	21,119,667	11,611,974	54.98	57.82	95.74	183,007	1.58
VM	1-2A	22,114,004	12,143,219	54.91	56.77	95.38	138,485	1.14
	1-2B	18,349,426	11,755,667	64.07	59.33	95.43	149,491	1.27
	1-10C	21,806,977	12,516,351	57.40	59.01	95.38	151,753	1.21
	2-1B	19,896,356	12,452,179	62.59	60.17	95.47	144,622	1.16
	2-9C	20,865,121	8,480,471	40.64	52,52	95.53	50,970	0.60

GC, guanine and cytosine content; Q30, quality score that 1 call in 1000 is predicted to be incorrect. Fish code indicates RAS unit (1 or 2), tank (1, 2, 6, 7, 9, or 10), and fish (A, B, C, or D) from which each blood plasma sample was obtained.

**Table 5:** Name, sequence, expression, fold change, and *q*-value of differentially expressed miRNAs from blood plasma of rainbow trout fed with fish meal (FM) and vegetable meal (VM) diets.

Name	Sequence	Mean RPM in VM Group	Mean RPM in FM Group	Log2 (Fold Change)	*q* -Value
omy-miR-730a-5p	uccucauugugcaugcugugug	14.45	1.41	+3.36	0.047
omy-miR-135c-5p	uauggcuuuuuauuccuacguga	23.53	2.45	+3.26	0.018
omy-miR-93a-3p	acugcaaaaccagcacuuccug	18.52	5.77	+1.68	0.047
omy-miR-152-5p	caaguucugugauacacuuaggcu	14.42	6.13	+1.23	0.036
omy-miR-133a-5p	agcugguaaaauggaaccaaa	87.99	37.60	+1.22	0.019
omy-miR-196a-2-3p	cuacaacacgaaacugucuga	32.88	14.76	+1.15	0.037

RPM, reads per million; n = 5.

**Table 6:** List of Gene Ontology (GO) biological process overrepresented by predicted mRNAs targeted by differentially expressed (DE) miRNAs.

GO Slim Biological Process	Nº of Genes	Fold Enrichment	FDR
Telomere maintenance (GO:0000723)	9	+6.93	8.65 × 10^−3^
Telomere organization (GO:0032200)	9	+6.77	8.49 × 10^−3^
Chromosome organization (GO:0051276)	37	+2.42	2.42 × 10^−3^
Organelle organization (GO:0006996)	124	+1.70	6.98 × 10^−5^
Cellular component organization (GO:0016043)	178	+1.49	9.83 × 10^−5^
Cellular component organization or biogenesis (GO:0071840)	180	+1.44	4.32 × 10^−4^
Cellular process (GO:0009987)	551	+1.17	8.81 × 10^−5^
Macromolecule metabolic process (GO:0043170)	207	+1.29	2.65 × 10^−2^
Nucleic acid metabolic process (GO:0090304)	71	+1.61	4.76 × 10^−2^
Nitrogen compound metabolic process (GO:0006807)	228	+1.26	3.49 × 10^−2^
Regulation of microtubule-based process (GO:0032886)	14	+3.77	1.89 × 10^−2^
Morphogenesis of an epithelium (GO:0002009)	31	+2.12	4.80 × 10^−2^
Tissue development (GO:0009888)	76	+1.68	8.60 × 10^−3^
Anatomical structure development (GO:0048856)	201	+1.43	1.92 × 10^−4^
Developmental process (GO:0032502)	203	+1.39	6.48 × 10^−4^
Anatomical structure morphogenesis (GO:0009653)	111	+1.69	1.67 × 10^−4^
Plasma membrane bounded cell projection organiz. (GO:0120036)	53	+1.85	1.28 × 10^−2^
Cell projection organization (GO:0030030)	56	+1.89	6.32 × 10^−3^
Intracellular transport (GO:0046907)	51	+1.77	4.85 × 10^−2^
Localization (GO:0051179)	182	+1.46	2.23 × 10^−4^
Establishment of localization (GO:0051234)	138	+1.44	8.85 × 10^−3^
Transport (GO:0006810)	138	+1.47	3.88 × 10^−3^
Cell development (GO:0048468)	71	+1.63	3.44 × 10^−2^
Cell differentiation (GO:0030154)	114	+1.54	4.78 × 10^−3^
Cellular developmental process (GO:0048869)	116	+1.55	2.50 × 10^−3^
Nervous system development (GO:0007399)	88	+1.61	9.00 × 10^−3^
System development (GO:0048731)	170	+1.49	1.47 × 10^−4^
Multicellular organism development (GO:0007275)	185	+1.43	4.36 × 10^−4^
Multicellular organismal process (GO:0032501)	213	+1.37	8.97 × 10^−4^
Animal organ development (GO:0048513)	122	+1.48	8.38 × 10^−3^
Negative regulation of apoptotic process (GO:0043066)	1	−0.08	2.41 × 10^−2^
Regulation of apoptotic process (GO:0042981)	5	−0.25	4.76 × 10^−2^

**Table 7:** List of Panther protein classes overrepresented by predicted mRNAs targeted by differentially expressed (DE) miRNAs from blood plasma of rainbow trout fed the VM diet when compared to those fed with the FM diet.

GO Slim Biological Process	Nº of Genes	Fold Enrichment	FDR
Microtubule binding motor protein (PC00156)	12	+5.62	1.02 × 10^−3^
Microtubule or microtubule-binding cytoskeletal protein (PC00157)	19	+2.49	2.48 × 10^−2^
ATP-binding cassette (ABC) transporter (PC00003)	9	+5.49	4.53 × 10^−3^
Membrane traffic protein (PC00150)	36	+2.23	2.16 × 10^−3^
Immunoglobulin receptor superfamily (PC00124)	0	−0.01	1.24 × 10^−2^
Defense/immunity protein (PC00090)	2	−0.13	3.46 × 10^−3^

## Data Availability

The experimental data are available upon request.

## Supplementary Materials

The following supporting information can be downloaded at: https://www.mdpi.com/article/10.3390/biology12070937/s1, Figure S1: Bile secretion KEGG pathway. Red boxes indicate predicted mRNA targets of the DE miRs found up-regulated in blood plasma from rainbow trout fed VM diet versus FM diet. BSEP, *bile salt export pump corresponding to ATP-binding cassette sub-family B member 11 a* (*abcb11a*); MRP2, *multidrug resistance-associated protein 2 corresponding to ATP-binding cassette sub-family C member 2* (*abcc2*); MRP3, *multidrug resistance-associated protein 3 corresponding to ATP-binding cassette sub-family C member 3* (*abcc3*); BCRP, *breast cancer resistance protein corresponding to ATP-binding cassette sub-family G member 2 b* (*abcg2b*). Modified from Kanehisa laboratories 2020; Table S1. Total variance explained by components of the principal component analysis (PCA); Table S2: Risk of hemolysis in blood plasma samples from rainbow trout fed with fish meal (FM) and vegetable meal (VM) diets; Table S3: List of identified circulating miRs in blood plasma; Table S4: List of potentially targeted mRNAs by DE miRs.
