# Supplementary material for: Specific Blood Plasma Circulating miRs Are Associated with the Physiological Impact of Total Fish Meal Replacement with Soybean Meal in Diets for Rainbow Trout (Oncorhynchus mykiss)
Source: Biology (Basel). 2023 Jun 30;12(7):937. doi: 10.3390/biology12070937 (PMC10376541; doi:10.3390/biology12070937)

Supplementary Figure S1. Bile secretion KEGG pathway. Red boxes indicate predicted mRNA targets of the DE miRs found up-regulated in blood plasma from rainbow trout fed VM diet versus FM diet. BSEP, *bile salt export pump* corresponding to *ATP-binding cassette sub-family B member 11 a* (*abcb11a*); MRP2, *multidrug resistance-associated protein 2* corresponding to *ATP-binding cassette sub-family C member 2* (*abcc2*); MRP3, *multidrug resistance-associated protein 3* corresponding to *ATP-binding cassette sub-family C member 3* (*abcc3*); BCRP, *breast cancer resistance protein* corresponding to *ATP-binding cassette sub-family G member 2 b* (*abcg2b*). Modified from Kanehisa laboratories 2020.

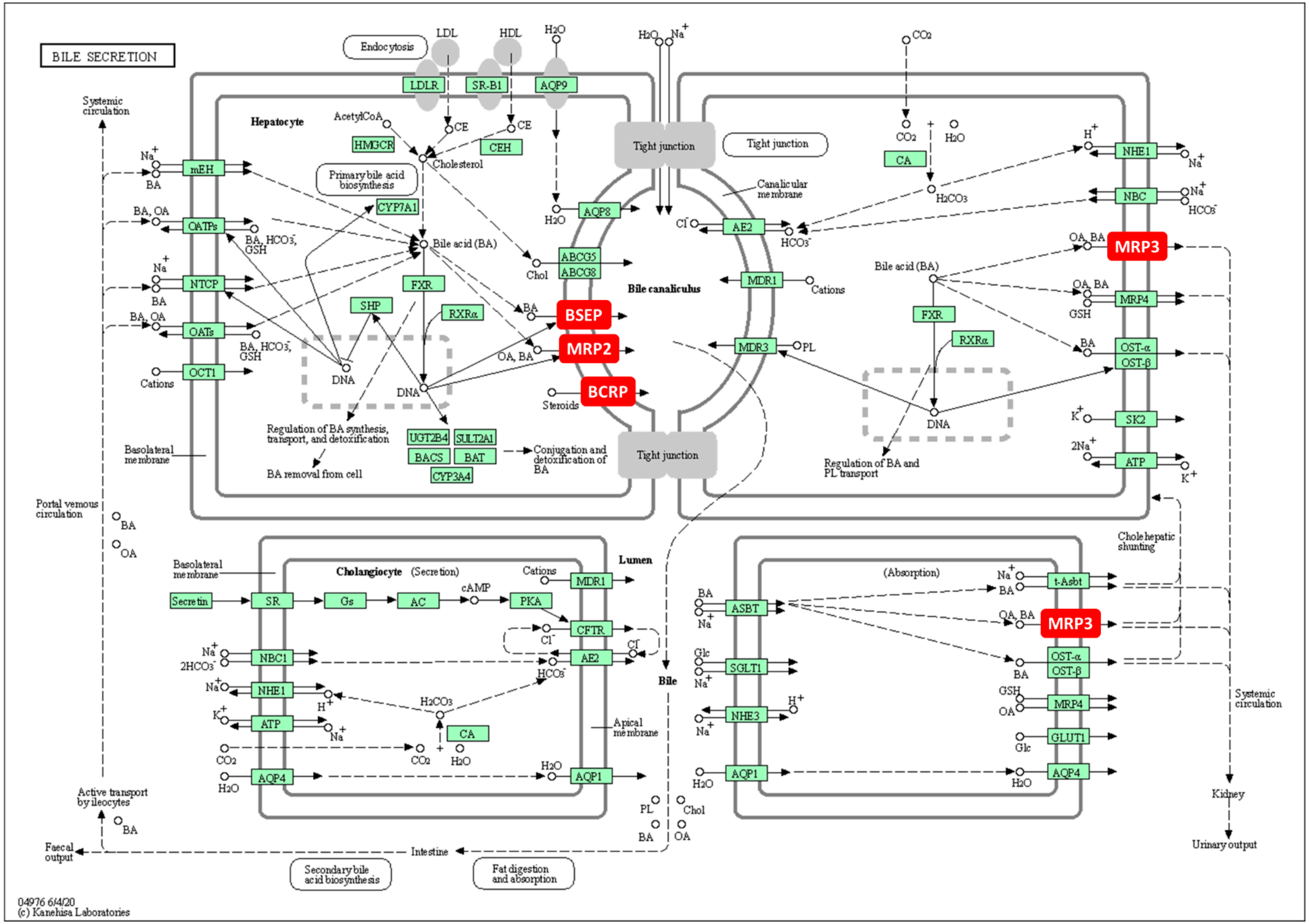

Supplement: Supplementary file 1 [file biology-12-00937-s001.zip › Supplementary Figure S1-The physiological impact.pdf]
