# Supplementary material for: Specific Blood Plasma Circulating miRs Are Associated with the Physiological Impact of Total Fish Meal Replacement with Soybean Meal in Diets for Rainbow Trout (Oncorhynchus mykiss)
Source: Biology (Basel). 2023 Jun 30;12(7):937. doi: 10.3390/biology12070937 (PMC10376541; doi:10.3390/biology12070937)
Supplement: Supplementary file 1 [file biology-12-00937-s001.zip › Supplementary Table S1-The physiological impact.pdf]

Supplementary Table S1. Total variance explained by components of the principal component analysis (PCA).

Component	Initial characteristic value			Extraction sums of squared loadings		
	Total	Variance (%)	Sum (%)	Total	Variance (%)	Sum (%)
**1**	12.20	40.67	40.67	12.20	40.67	40.67
**2**	5.66	18.89	59.56	5.66	18.89	59.56
**3**	4.32	14.41	73.98			
**4**	3.05	10.16	84.15			
**5**	2.33	7.79	91.94			
**6**	1.48	4.95	96.90			
**7**	0.93	3.10	100.00			
**8**	1.0×10^{-13}	1.0×10^{-13}	100.00			
**9**	1.0×10^{-13}	1.0×10^{-13}	100.00			
**10**	1.0×10^{-13}	1.0×10^{-13}	100.00			
**11**	1.0×10^{-13}	1.0×10^{-13}	100.00			
**12**	1.0×10^{-13}	1.0×10^{-13}	100.00			
**13**	1.0×10^{-13}	1.0×10^{-13}	100.00			
**14**	1.0×10^{-13}	1.0×10^{-13}	100.00			
**15**	1.0×10^{-13}	1.0×10^{-13}	100.00			
**16**	1.0×10^{-13}	1.0×10^{-13}	100.00			
**17**	1.0×10^{-13}	1.0×10^{-13}	100.00			
**18**	1.0×10^{-13}	1.0×10^{-13}	100.00			
**19**	1.0×10^{-13}	1.0×10^{-13}	100.00			
**20**	-1.0×10^{-13}	-1.0×10^{-13}	100.00			

**21**	$-1.0x10^{-13}$	$-1.0x10^{-13}$	100.00
**22**	$-1.0x10^{-13}$	$-1.0x10^{-13}$	100.00
**23**	$-1.0x10^{-13}$	$-1.0x10^{-13}$	100.00
**24**	$-1.0x10^{-13}$	$-1.0x10^{-13}$	100.00
**25**	$-1.0x10^{-13}$	$-1.0x10^{-13}$	100.00
**26**	$-1.0x10^{-13}$	$-1.0x10^{-13}$	100.00
**27**	$-1.0x10^{-13}$	$-1.0x10^{-13}$	100.00
**28**	$-1.0x10^{-13}$	$-1.0x10^{-13}$	100.00
**29**	$-1.0x10^{-13}$	$-1.0x10^{-13}$	100.00
**30**	$-1.0x10^{-13}$	$-1.0x10^{-13}$	100.00
