# Supplementary material for: Specific Blood Plasma Circulating miRs Are Associated with the Physiological Impact of Total Fish Meal Replacement with Soybean Meal in Diets for Rainbow Trout (Oncorhynchus mykiss)
Source: Biology (Basel). 2023 Jun 30;12(7):937. doi: 10.3390/biology12070937 (PMC10376541; doi:10.3390/biology12070937)
Supplement: Supplementary file 1 [file biology-12-00937-s001.zip › Supplementary Table S2-The physiological impact.pdf]

Supplementary Table S2. Risk of hemolysis in blood plasma samples from rainbow trout fed with fish meal (FM) and vegetable meal (VM) diets.

Experimental group	Fish code	Absorbance*
FM	1-6B	0.34
	1-9B	0.29
	1-9C	0.29
	2-7A	0.25
	2-10C	0.20
VM	1-2A	0.25
	1-2B	0.31
	1-10C	0.20
	2-1B	0.36
	2-9C	0.32

* *Absorbance at λ=414 nm*
